# Seasonal variation in δ^13^C of *Pinus. yunnanensis* and *Pinus. armandii* at different stand ages

**DOI:** 10.1038/s41598-023-34920-3

**Published:** 2023-05-16

**Authors:** Yuanxi Liu, Junwen Wu, Danzi Wu, Shiming Li, Lina Wang

**Affiliations:** College of Forestry, Southwest Forestry University, Kunming, 650224 Yunnan China

**Keywords:** Hydrology, Ecophysiology, Stable isotope analysis

## Abstract

Seasonal drought is common in Yunnan province, and water is the dominant factor limiting the growth of *Pinus. yunnanensis* and *Pinus. armandii*. The water use efficiency (WUE) of the two species is poorly understood. Needles were collected in a plantation (*P. yunnanensis* and *P. armandii* mixed forest) in four seasons, and the needle δ^13^C values were measured. The selected species had larger δ^13^C values and exhibited higher WUE than typical subtropical species. *P. armandii* needles showed a more conservative water use strategy (high WUE) than *P. yunnanensis*. There were significant differences in the δ^13^C values of *P. armandii* between the two ages, whereas no difference was observed in the δ^13^C values of *P. yunnanensis*. The lowest δ^13^C value in the young *P. armandii* forest was observed in spring, whereas the δ^13^C value of middle-aged forests did not differ between the seasons. The δ^13^C value of young *P. yunnanensis* forests showed no difference in the four seasons, and the maximum value was observed in summer in middle-aged forests. In general, the δ^13^C value of *P. armandii* was lowest in spring, whereas that of *P. yunnanensis* was higher in spring and winter. The needle δ^13^C values were lower in spring and winter, indicating that the season had different effects on the δ^13^C values of different tree species. Correlation analysis between the needle δ^13^C values and meteorological data indicated that temperature and precipitation were the dominant factors affecting WUE in *P. yunnanensis* and *P. armandii*. The effect of temperature on WUE was greater in *P. yunnanensis* middle-aged forests. The identification and selection of subtropical tree species with high WUE are critical to maintaining high levels of forest benefits under limited water conditions.

Water limitation may become the main reason for limiting plant productivity due to the intensification of global climate change. Therefore, improving plant water use efficiency (WUE) is a critical research goal in the future. WUE reflects the coupling of the carbon and water cycles between plants, soil, and the atmosphere. Investigating WUE can improve our understanding of the coupling mechanism of the carbon and water cycles of terrestrial ecosystems^1,2^. Research on WUE has been conducted at different scales, including leaf^3,4^, canopy^5^, single plant^6^, stand^7^, community^8^, ecosystem^9^, and landscape scales^10^. Most studies have focused on the leaf scale^11^, because WUE on the leaf scale can reveal the internal water use mechanism of plants and is the basis for WUE on a larger scale. Gas exchange and stable carbon isotope methods have been typically used to assess plant WUE at the leaf scale^1^. The gas exchange method represents the behavior of plant leaves in a specific period; thus, this method is suitable for studying the physiological and ecological processes causing rapid changes in WUE. Since plant photosynthesis is highly sensitive to environmental conditions, the photosynthetic rate may not be related to plant WUE and can only be used to explain plant production and responses to environmental factors. The daily variation of WUE under specific conditions does not represent the long-term WUE in a changing environment^12^. Therefore, it is necessary to use other methods to study long-term plant WUE. The stable carbon isotope method is highly effective for analyzing the nutrient cycle of ecosystems and has been used to assess plant WUE to understand and predict plant responses and adaptations of forest vegetation to global change^13^.

The stable carbon isotope ratio of plant leaves (δ^13^C) is a suitable index for evaluating long-term plant WUE. Leaves are critical photosynthetic organs of plants for carbon and water exchange. The δ^13^C values of leaves are highly sensitive to environmental changes and have been typically used to study the response of plants to climate and environmental conditions. They reflect the leaves’ photosynthetic and transpiration characteristics and WUE, the plant’s adaptation to the environment and responses to environmental changes, and the characteristics of the climate and environment, such as precipitation^14^, soil moisture content^4,15–17^, irradiance gradient^18^, elevation^19^, age^18^, seasonal variation^20–22^, and heavy metal pollution^23^. Scholars have performed extensive research on plant WUE and its influencing factors. For example, it was found that WUE was significantly positively correlated with temperature, annual rainfall, and leaf area index and significantly negatively correlated with effective photosynthetic radiation^9^. However, due to the diversity of vegetation types and the complexity of WUE, the dynamic changes in short-term WUE and the plants’ response to environmental factors are not well known. There is no clear understanding of the main factors affecting short-term WUE and its mechanism, and the contribution of the main factors has not been quantified^2,9^. In past studies, a direct relationship between plant leaf WUE and tree age has led to two opposing conclusions, the first being that plant leaf WUE decreases with increasing tree age^42–44^, leading to a natural partitioning of carbon stable isotopes that is changing during growth. The other is that plant leaf WUE increases with increasing tree age^45^, resulting from the influence of plant stomatal conductance on plant photosynthesis. There is still no unified conclusion about the factors influencing plant leaf WUE.

Yunnan province is located on the southwest border of China and has a unique geographical location, complex terrain and landform, and a monsoon climate. It is located on a plateau, has distinct dry and wet seasons, and experiences high-frequency and long-duration winter and spring drought in central area of Yunnan province. These natural disasters affect the social and economic development of Yunnan province. Medium and severe droughts have shown an increasing trend^24^. Thus, the selection of appropriate tree species that can withstand water shortages is a crucial goal of forest management. A clear understanding of the water-use characteristics of various tree species is required, and WUE is an objective index to evaluate the water use of plants and their drought resistance.

*Pinus. yunnanensis* and *Pinus. armandii* are endemic tree species that dominate forests in southwest China. *P. yunnanensis* grows at an altitude of 1500–2500 m, and *P. armandii* is commonly found at 1629–3117 m. *P. yunnanensis* is a dominant species in the forest community because it prefers high light conditions and is tolerant of barren areas and low temperatures. *P. yunnanensis* forests cover about 500 × 104 hm^2^ in Yunnan Province, accounting for about 52% of the forest area. The two species provide important ecological, economic, and social benefits to the region and have strong ecological adaptability and significant research value. Many studies have focused on the canopy and soil seed banks for regeneration^25^, seed germination^26,27^, intra-specific competition^28,29^, secondary forest succession^30^, and forest management^30–32^. The WUE of *P. yunnanensis* and *P. armandii* is poorly understood. This study analyzes the seasonal variation in the δ^13^C values and the influence of meteorological factors to understand the water use strategy of the two coniferous species and provide information on species selection. The objectives are to (1) investigate the seasonal variation in the needle δ^13^C values of *P. yunnanensis* and *P. armandii* plantations with different stand ages and (2) determine the relationship between the needle δ^13^C values and meteorological factors. (3) To elucidate how these two tree species adapt to seasonal drought in central area of Yunnan province from the perspective of WUE.

## Materials and methods

### Study area

**Figure 1:**
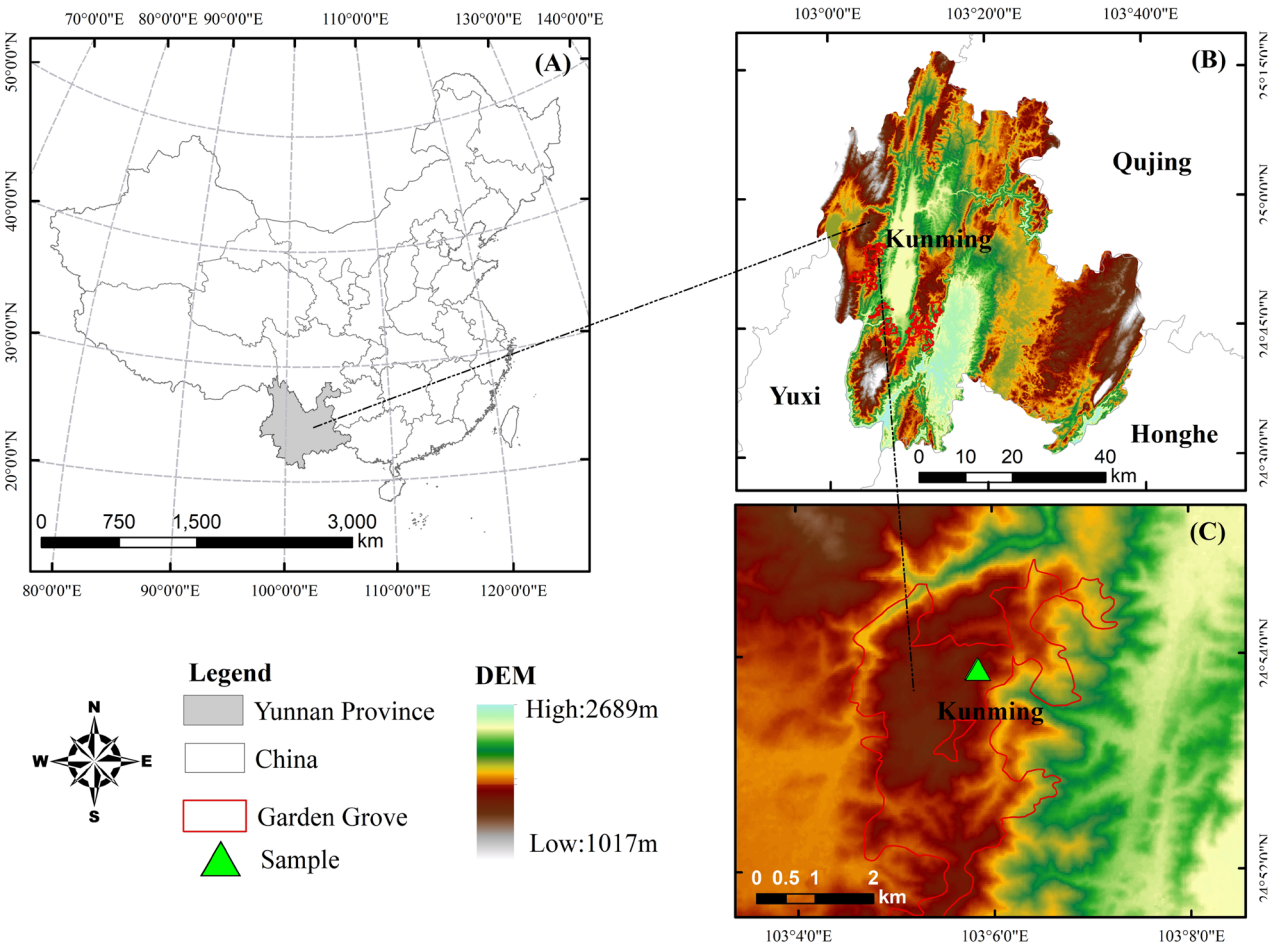
Map of research site Note: maps and satellite imagery were generated using the ArcGIS10.8 sofware from American ESRI Company. https://support.esri.com/en/Products/Desktop/arcgis-desktop/arcmap.

Yiliang County is subordinate to Kunming City, Yunnan Province. It is located in central area of Yunnan province (24°39′–25°17′ N and 102°58′–-03°28′ E. The standard sample plots at the Huayuan forest farm in Yiliang County are located at 24°54′–25°00′ N, 103°00′–103°30′ E at 1300–2800 m above sea level (Fig. 1, and Figure 1 is done in Arcgis 10.8 software). The area has a subtropical monsoon climate that is dry in winter and spring and wet in summer. There is no severe cold in winter, and summers are hot. The average annual rainfall in this area is 912.2 mm (80% of precipitation occurs between June and October), the annual average temperature is 16.3 ℃, and the sunshine hours are 2177.3 h, The average annual frost-free period is 260 d, and the soil is predominantly red soil.

### Sample site selection and sampling

We analyzed the differences in δ^13^C values between *P. yunnanensis* and *P. armandii* needles at different ages and in different seasons. We used the "time-substituted space" method commonly used in forest ecology research to select sample plots to study the differences between different forest ages. Replicates of sample plots and trees were performed in accordance with the relevant guidelines and regulations. The first needle collection was carried out in early January 2021. Subsequently, needles were collected once in each season (January, May, August, and November). Two stands of mixed plantations (*P. yunnanensis* and *P. armandii*) were selected, and standard plots were established in representative locations with low disturbances. The topographic information of each sample site is shown in Table 1. The plots were rectangular (30 m × 30 m), and their boundaries were measured using a compass meter (closure difference 1/200). The data were collected in the plots, and a buffer zone of 5 m was left between the sample plots. The sample plots were located on the upper and middle slopes, and three trees were selected in each plot as standard trees. The needles were collected from different branches for each age class and combined in a replicate sample. The samples were washed three times with pure deionized water, heated in an oven at 70 °C for 48 h to a constant weight, ground, and crushed in an agate mortar. Subsequently, the material was sieved through a 0.125 mm (120 mesh) sieve and packed in tin foil bags for the determination of the δ^13^C values.

**Table 1:** Sample sites information.

Sample Sites	Age	Elevation (m)	Longitude and latitude	Slope position and direction
1	Middle-aged forests	2346.0	24˚54′4″N 103˚5′9″E	Mid-slope Due south169˚ Half-Yang Slope
2	Young forests	2389.0	24˚54′3″N 103˚5′9″E	Uphill position Due west 267˚ Sunny slope
3	Middle-aged forests	2319.0	24˚53′48″N 103˚5′9″E	Mid-slope Due east 95˚ Half-sunny slope
4	Young forests	2369.0	24˚53′48″N 10˚5′38″E	Mid-slope north of east 39˚ Half-sunny slope
5	Middle-aged forests	2335.0	2453′48″N 103˚5′37″E	Mid-slope south of west 232˚ Half shaded slope
6	Young forests	2299.0	24˚53′49″N 103˚5′33″E	Downhill position Due north 6˚ Half-sunny slope

### Plant tissue analysis

Determination of stable carbon isotope natural abundance: the carbon stable isotope composition of leaves is determined by the stable isotope proportional mass spectrometer (DELTA V Advantage, USA) (δ^13^C, ‰) is expressed as follows:

1 $$ \delta^{{{13}}} {\text{C}} = \, \left( {{\text{R}}_{{{\text{sam}}}} /{\text{R}}_{{{\text{std}}}} - {1}} \right) \, \times { 1}000 $$

where δ^13^C is the carbon isotope value of the sample, R_sam_ and R_std_ are the ratios of the heavy and light isotopic abundances of the elements in the sample and the international standard, respectively (^13^C/^12^C). Determination accuracy: δ^13^C: ±  < 0.1 ‰ (non-labeled samples).

Principle: The sample was heated at a high temperature in the elemental analyzer to produce CO_2_, and the mass spectrometer was used to calculate the δ^13^C value of the sample based on the ^13^C to ^12^C ratio of CO_2_ and a comparison with an international standard (Pee Dee Belemnite (PDB)) as follows^33^.

### Climate data

Meteorological data were obtained from the National Meteorological Information Center-China Meteorological Data (cma.cn) based on the geographical coordinates of the sampling points. The dataset contains daily rainfall and temperature, monthly average air pressure, monthly average relative humidity, monthly average surface temperature, monthly average evapotranspiration, monthly average sunshine hours, and average wind speed for Yiliang County for 2021.

### Statistical analyses

All statistical analyses were performed using SPSS version 24.0 for Windows (SPSS Inc., Chicago, IL, USA). Fixed effects were stand age, season, species, season, and their interaction. The normal distribution of the errors and homogeneity of variance were assessed, and data with residuals not conforming to the assumptions were log-transformed. The data are presented as means ± standard error (SE) for different treatments. ANOVA was used to analyze the differences between the means, and Tukey’s tests were used for post-hoc comparisons between groups. Graphpad Prism 8.0 and Origin 8.0 were used to draw the graphs. Pearson's method was used to analyze the correlation between the δ^13^C values of needles and meteorological factors of *P. yunnanensis* and *P. armandii* in different forest ages.The significance level was *P* = 0.05.

## Results

### Seasonal variation of precipitation and temperature

**Figure 2:**
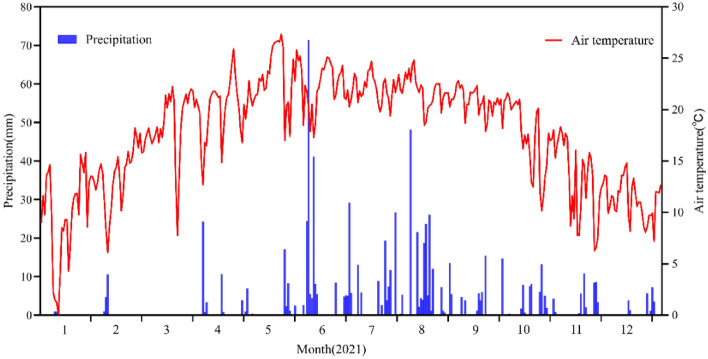
Daily air temperature and daily precipitation during 2021.

Figure 2 shows the daily fluctuations in rainfall and temperature at the study site in 2021. The total annual precipitation was 802.6 mm, with 80% occurring during the growing season (June to October) (Fig. 2). The highest monthly precipitation occurred in August, and the lowest occurred in March. Relatively high rainfall amounts and high-frequency rainfall events occurred between June and August. Conversely, the period from November to May had relatively low rainfall, especially from December to March, and a low frequency of rainfall events (Fig. 2). Thus, in the Yiliang County of Yunnan, plants must withstand a significant 7-month continuous winter-spring drought, during which the total annual rainfall is only 10–20% of the total rainfall. In addition, the mean annual temperature in the study area was 10.03 °C, with the highest temperatures occurring in May and the lowest in January. Relatively high temperatures occurred between March and October, and relatively low temperatures were observed in November, December, January, and February. Therefore, we believe that the plants did not experience moisture stress in June and October but suffered from severe water deficits from November to May. The area experienced a large rainfall event in the third sampling period in August, and the fourth sampling period in November was the end of the rainy season when the area entered a period of winter drought with a few small rainfall events.

### Effects of different factors on needle δ13C values

The effects of different tree species (*P. yunnanensis*, *P. armandii*), different seasons (January, May, August, November), different age classes, and their interactions on the δ^13^C values are listed in Table 2. The tree species and season had a highly significant effect (*P* < 0.01), age class had a significant effect (*P* < 0.05), and the interaction between tree species and age class had a significant effect on δ^13^C (*P* < 0.05). However, the interaction between tree species and season and season and age did not have a significant effect on δ^13^C.

**Table 2:** Effects of different factors and their interaction on needle δ^13^C in *P. armandii* and *P. yunnanensis.*

Index	Factor	*F*
δ^13^C	Tree species	17.656**
	Season	4.832**
	Stand age	6.667*
	Tree species × Season	2.003
	Tree species × Stand age	4.555*
	Season × Stand age	0.641

*indicates a significant difference (*P* < 0.05) and **indicates a highly significant difference (*P* < 0.01).

### Effect of tree species on δ13C

**Figure 3:**
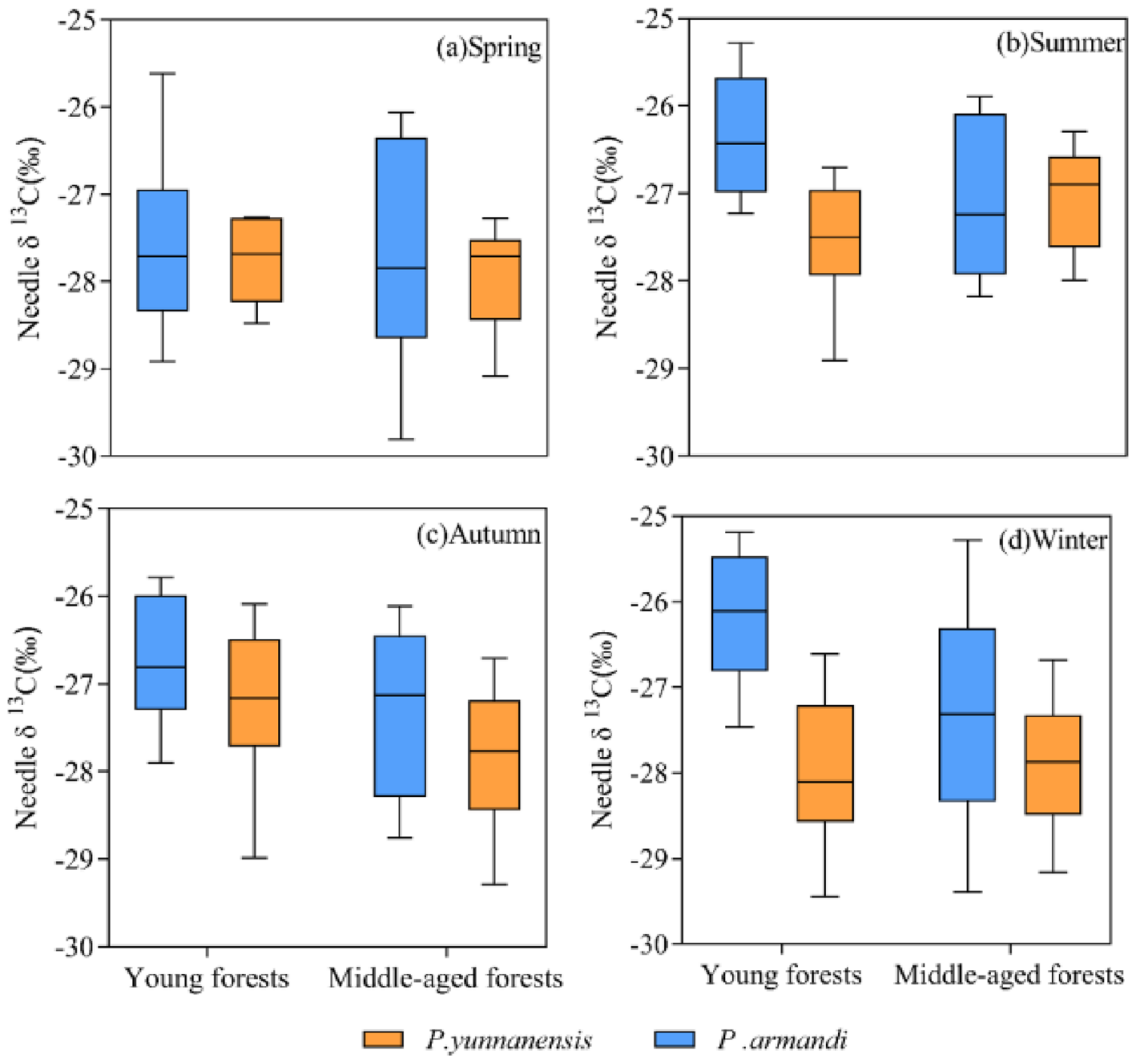
Boxplots of needle δ^13^C values of different tree species and stand ages in different seasons.

There was no significant difference in the needle δ^13^C between *P. armandii* and *P. yunnanensis* in middle-aged stands (*t* = 1.326, *P* < 0.01) and young stands (*t* = 4.535, *P* < 0.01). With the change of seasons, the needle δ^13^C values of *P. armandii* young forests were significantly higher than those of *P. yunnanensis* young forests, by 0.67%, 4.23%, 1.92% and 6.38%, respectively; the needle δ 13C values of *P. armandii* middle-aged forests were higher than those of *P. yunnanensis* middle-aged forests, by 0.60%, − 0.20%, 1.96% and 1.92%, respectively (Fig. 3).

### Effect of tree age on δ13C

**Figure 4:**
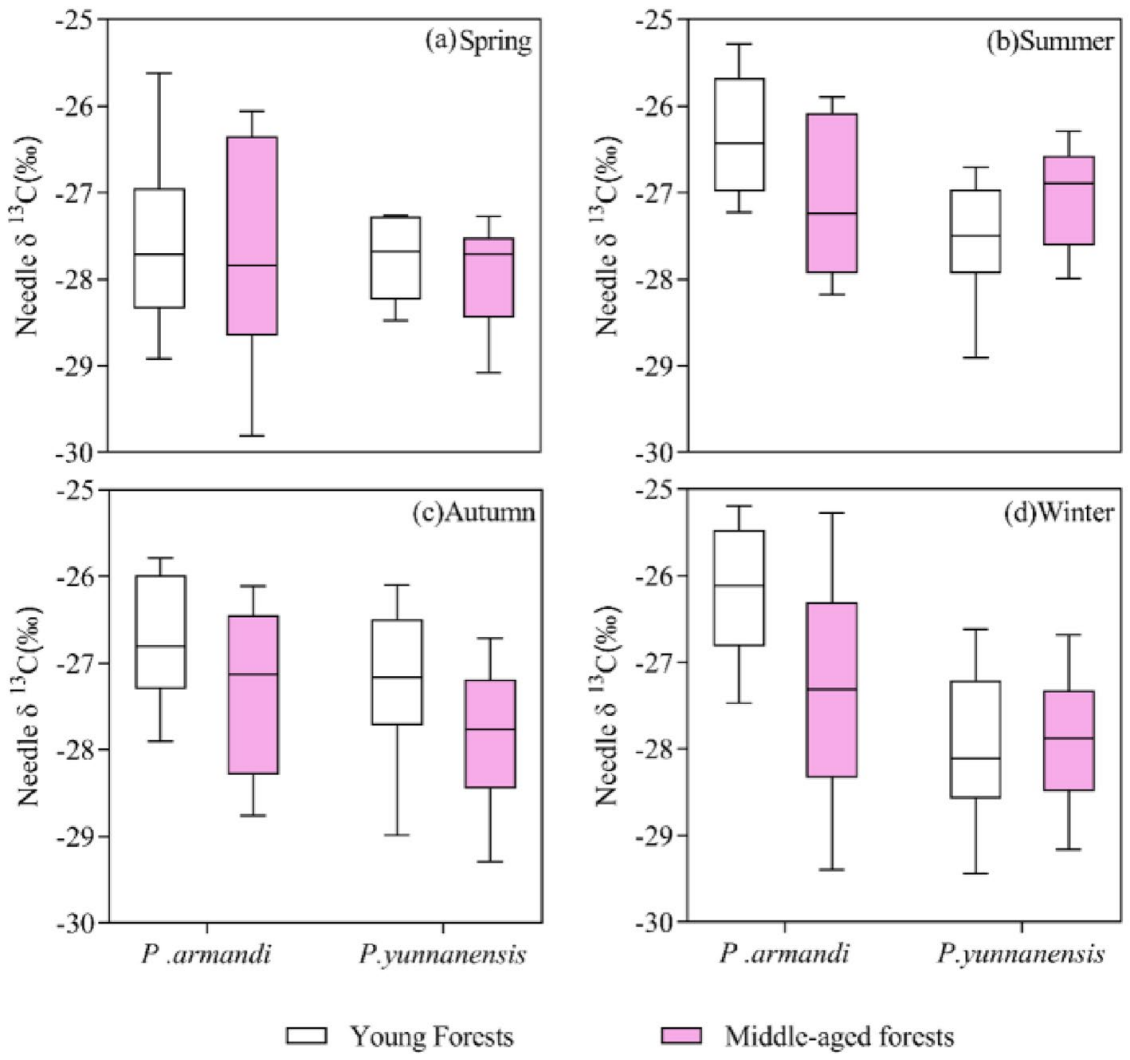
Effect of stand age on δ^13^C.

There was a highly significant difference in the needle δ^13^C between different age classes of *P. armandii* (*t* = 2.805, *P* < 0.01) but not between different age classes of *P. yunnanensis* (*t* = 0.359, *P* > 0.05). The δ^13^C values of *P. yunnanensis* were slightly higher in young forests than in middle-aged forests in the spring (0.67% higher) and autumn (2.16% higher). In contrast, the δ^13^C values were slightly lower in young forests than in middle-aged forests in summer (1.70% lower) and winter (0.25% lower) (Fig. 4).

### Effect of season on needle δ13C

**Figure 5:**
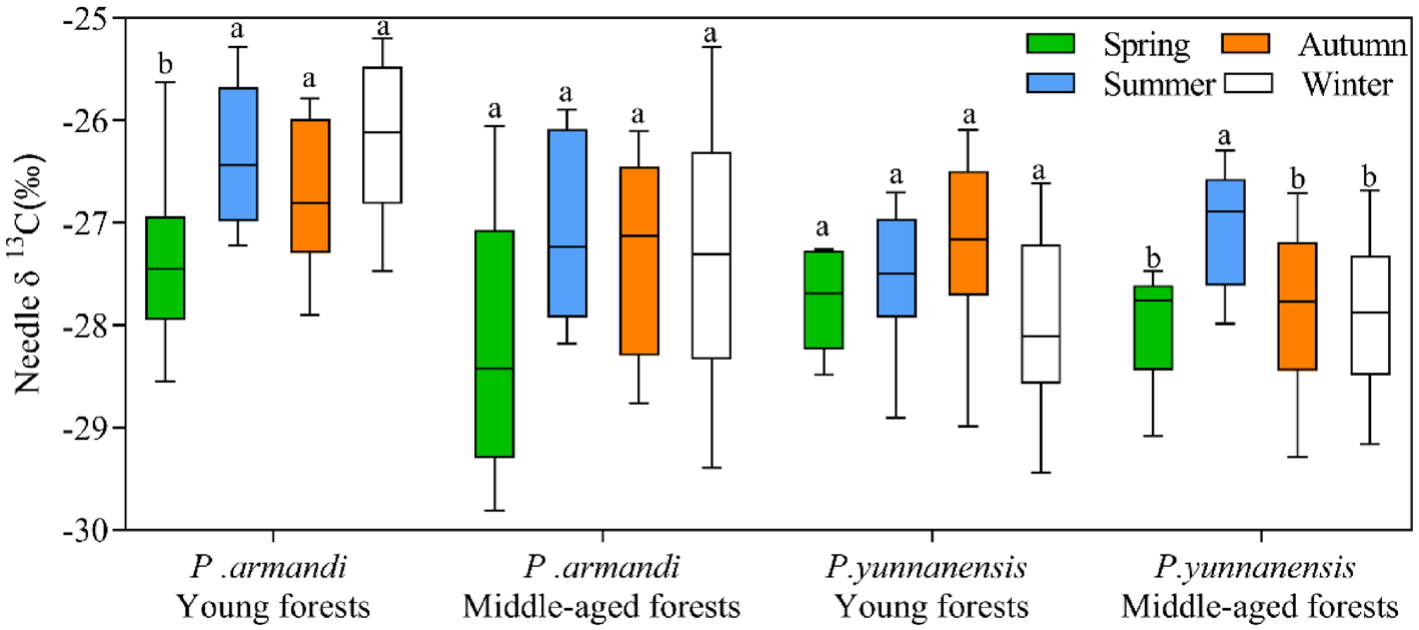
Seasonal variation of δ^13^C in coniferous leaves of different tree species in different aged stands. Different lowercase letters indicate significant differences between different drought stress treatments (*P* < 0.05).

The needle δ^13^C values were significantly higher in summer, autumn, and winter than in spring in young stands of *P. armandii*, whereas the δ^13^C values in middle-aged stands of *P. armandii* did not differ significantly between the four seasons (Fig. 5). The δ^13^C values in young *P. yunnanensis* forests did not differ significantly between the four seasons, whereas those of middle-aged *P. yunnanensis* forests were significantly higher in summer than in spring, autumn, and winter. This result indicates that *P. armandii* has higher needle δ ^13^C values in summer, autumn, and winter and lower values in spring, whereas *P. yunnanensis* has higher needle δ^13^C values in summer and autumn and lower values in spring and winter.

### Relationship between δ13C and meteorological factors

**Figure 6:**
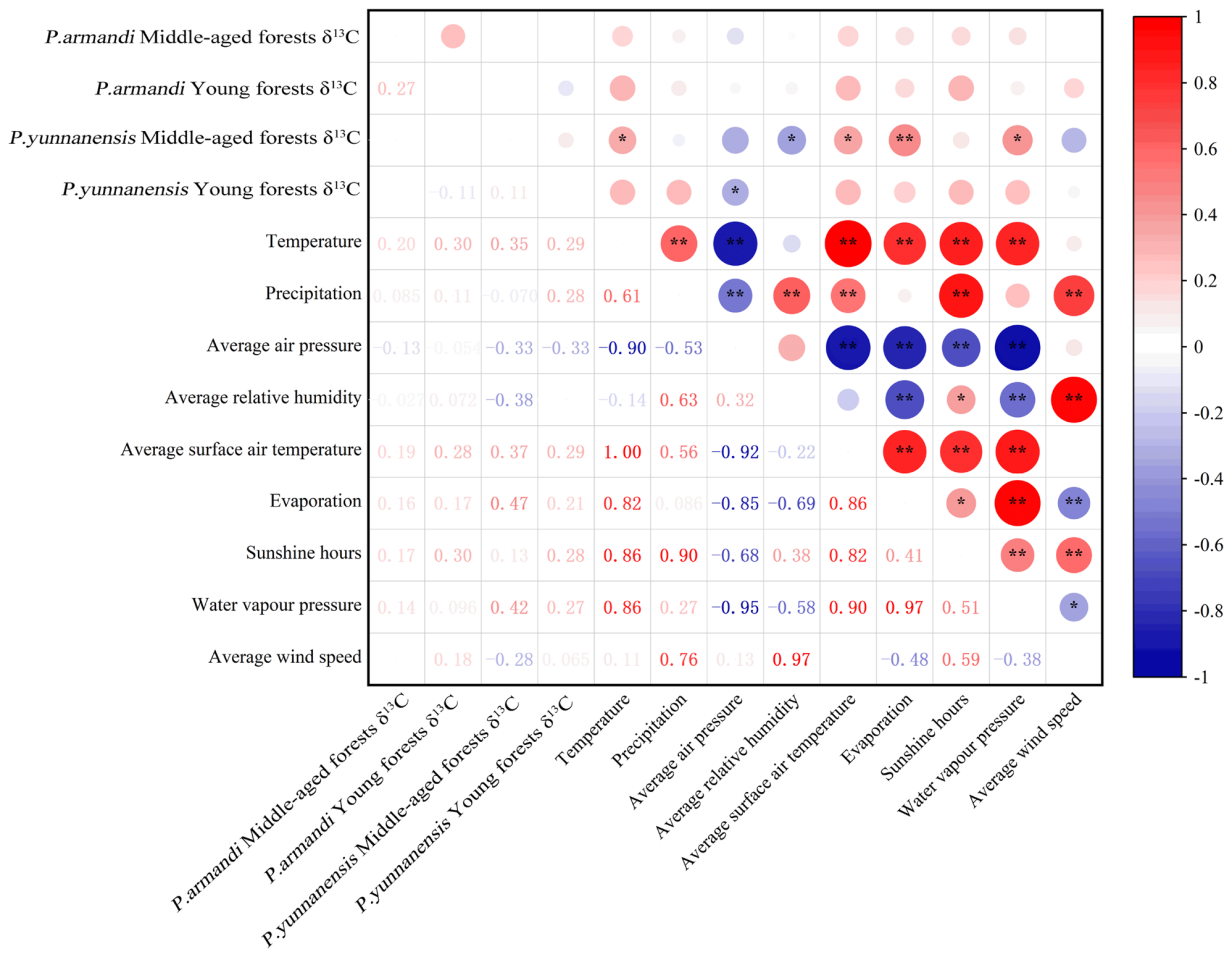
Correlation between stable carbon isotope values and meteorological factors. * indicates a significant difference (*P* < 0.05) and ** indicates a highly significant difference (*P* < 0.01).

Correlations were observed between the δ^13^C values of *P. yunnanensis* and the meteorological factors, and no significant correlations occurred between the δ^13^C of *P. armandii* and the meteorological factors (Fig. 6). The needle δ^13^C value was significantly correlated with evapotranspiration (*P* < 0.01), temperature, mean surface temperature, water vapor pressure (*P* < 0.05), and mean relative humidity (*P* < 0.05) in middle-aged *P. yunnanensis* forests. The needle δ^13^C value was only significantly correlated with mean air pressure (*P* < 0.05) in young *P. yunnanensis* forests.

## Discussion

### Needle δ13C values of different tree species

Plants with different photosynthetic pathways (C_3_ and C_4_) have different leaf δ^13^C values due to differences in primary carboxylases^34^. The δ^13^C values in C_3_ plants range from -20‰ to -35‰, whereas those in C_4_ plants range from − 7 to − 15‰^35^. *P. armandii* and *P. yunnanensis* are C_3_ plants (the δ^13^C values ranged from − 25.19 to − 29.81‰ in this study) (Fig. 5). These values are consistent with those of *Radermachera sinica*, *Sapium rotundifolium*, *Sterculia euosma*, *Schefflera octophylla*, *Alchornea trewioides,* and *Vitex negundo*, which grow on continuous dolomite surfaces in subtropical China^36^. These six C_3_ species had the same range of δ^13^C values^36^. The range of leaf δ^13^C values of C_3_ plants differs for different climatic zones^37^. Our study area has a subtropical monsoon climate with only 912.2 mm annual precipitation (Fig. 2), but *P. yunnanensis* and *P. armandii* needles have higher δ^13^C values than typical subtropical species (from − 31.1 to − 30.5^37^, indicating that both species exhibit high WUE. Guo et al.^38^ found that different tree species had significantly different δ^13^C values (Table 1). The *P. armandii* needles had a higher δ^13^C value than *P. yunnanensis*, indicating that *P. armandii* exhibited higher WUE than *P. yunnanensis*. This difference was attributed to differences in the genetic traits of the species, resulting in different strategies for adapting to environmental change.

### Leaf δ13C values of plants of different stand ages

The natural abundance of stable carbon isotopes in plant leaves changes during growth^39^. A comparative study of leaf δ^13^C values of^42^ grassland species in arid regions of the northwestern United States showed that the δ^13^C values of 1-year-old plants were lower than those of perennial plants^40^. Leaf δ^13^C analysis of several major warm-temperate deciduous tree and shrub species in Beijing's Dongling Mountains revealed a gradual decrease in the δ^13^C value from early to late growth^41^. In this study, stand age had a significant effect on the needle δ^13^C value (Table 1). The value was significantly higher in young stands than in old stands of *P. armandii*, especially in winter. The δ^13^C value was 4.30% higher in young stands than in middle-aged stands, indicating that a change occurred during growth. This result is consistent with the study by Zhang et al.^42^, who focused on different ages of *Populus euphratica Oliv* on the Tarim River. The WUE decreased with the increasing stand age. Kong et al.^43^ studied the WUE of different stand ages of *Cunninghamia lanceolata* (Lamb.) Hook plantations. The ranking of the WUE was current year > 1 year > 2 years > 3 years. The leaf δ^13^C values increased with the increasing stand age, which might be related to the extent of the root system. As the plant grows and matures, the root system expands, and its leaf δ^13^C decreases, resulting in low WUE. *P. sylvestris* showed similar characteristics, with older needles showing a gradual decline in water use^44^, whereas Casper et al.^45^ observed the opposite in *Cryptantha flava*. The WUE was significantly higher in mature stands than in younger stands, which was attributed to the fact that mature plants maintained a higher photosynthetic rate during the growing season, whereas stomatal conductance is similar to that of young plants.

This study showed no significant difference in the δ^13^C values of *P. yunnanensis* needles between young and middle-aged stands, suggesting that the δ^13^C values in *P. yunnanensis* needles are relatively stable during growth. This result is consistent with the findings of Kong^43^, who analyzed the factors influencing WUE in major forest types in the subtropics. The author stated that the photosynthetic rate of leaves was influenced by the resistance of the leaf pulp, limiting CO_2_ diffusion with increasing age^46^. Biochemical limitations exist due to changes in primary carboxylase activity and photochemical properties due to changes in photochemical properties^46^. A decrease in the photosynthetic rate and stomatal conductance of needles may indicate low WUE.

### δ13C values of plant leaves in different seasons

Water is the primary environmental factor limiting plant growth and development, and water deficiency or excess water can severely limit plant productivity. A survey of global conifer δ^13^C values showed that in the absence of a water deficit, the δ^13^C value reached an asymptotic value, precipitation and transpiration reached equilibrium, and stomatal conductance reached the maximum^47^. Under water stress conditions, the productivity of plants decreases. A study of *Quercus prinus* found that the leaf δ^13^C value was lower in autumn before the leaf drop than in the middle of summer^48^. Seasonal variations were observed in the δ^13^C value of four major shrub species (*Haloxylon ammodendron, Nitraria tangutorum Bobr, Calligonum mongolicum,* and *Tamarix ramosissima*). The relationships between the δ^13^C values and meteorological factors in an arid desert region were analyzed, and significant differences in the δ^13^C value were observed between different months and species^38^.

In this study, the needle δ^13^C values in young stands of *P. armandii* were significantly higher in summer, autumn, and winter than in spring, whereas there was no significant difference in middle-aged stands. The δ^13^C values in young stands of *P. yunnanensis* were not significantly different between the seasons, and those in middle-aged stands were significantly higher in summer than in spring, autumn, and winter. This result indicated that the needle δ^13^C values in young stands of *P. armandii* and middle-aged stands of *P. yunnanensis* were more sensitive to seasonal changes. The lower temperatures in spring and winter and the low amount of precipitation (less in spring than in winter) in this study (Fig. 2) may have contributed to the lower needle δ^13^C values in spring and winter in *P. yunnanensis* middle-aged forests, and the lower needle δ^13^C values in spring and higher values in winter in young *P. armandii* forests (Fig. 5).

Loader et al.^49^ suggested that higher temperatures are accompanied by an increase in plant photosynthetic capacity, resulting in higher WUE. The water status affects the water use of plants, and the transpiration rates are reduced to a greater extent than the photosynthetic rate when rainfall is insufficient. In addition, the partial closure of stomatal can increase WUE^40^. It is possible that the drop in temperature in winter, when the precipitation is lower than in spring, did not affect the *P. armandii* needle δ^13^C values. The plants reduced stomatal conductance when the moisture declined, resulting in a decrease in photosynthetic rate and carbon assimilation. Hence, the needle δ^13^C values and WUE were higher in young *P. armandii* forests^50^. In contrast, the *P. yunnanensis* middle-aged forests showed the same trend in winter and spring. The lower temperatures resulted in lower needle δ^13^C values in *P. yunnanensis* middle-aged forests, indicating that *P. yunnanensis* forests are more sensitive to temperature. This result is consistent with that of Lu et al.^51^, who studied WUE in oil pine. The relatively high temperature and precipitation in the summer and autumn growing season resulted in higher needle δ^13^C values and high water demand of *P. yunnanensis* and *P. armandii*. An increase in the temperature in the early growing season and the average growing season temperature accelerates plant transpiration and promotes plant photosynthesis. Our results suggest that the two species in the study area are affected by the combined effects of moisture and temperature, resulting in different WUE between *P. yunnanensis* and *P. armandii* during winter. The temperature had a larger effect on the WUE of *P. yunnanensis* middle-aged stands, whereas precipitation was the dominant factor affecting the WUE of young-aged *P. armandii* stands. This result is consistent with the findings of Zhou et al.^52^ for Acacia (*Robinia pseudoacacia* L.) in the Minquan, Henan, and Shanxi areas in China and Lu et al.^51^ for *P. tabuliformis* in the mountains of Beijing.

### Relationships between leaf δ13C and meteorological factors

Meteorological factors (e.g., sunlight, atmospheric pressure, temperature, precipitation, etc.) affect plant carbon isotopes by influencing plant leaf gas exchange activities^53^. The dominant climatic factors are light, moisture, and temperature 2. Worldwide, WUE was highest in temperate plants (leaf δ^13^C value: − 2.95 to − 2.62%), followed by subtropical plants (leaf δ^13^C value: − 3.11 to − 3.05) and tropical plants (leaf δ^13^C value: − 3.21 to − 3.18%)^2^. The temperature affects the activity of enzymes involved in photosynthesis, affecting plant carbon isotope fractionation^54^. Some studies have shown a significant negative correlation between temperature and plant δ^13^C value^55^, whereas other studies observed a positive correlation^49^.

In contrast to studies showing a significant negative correlation between temperature and plant leaf δ^13^C values^56^, Yuan et al.^57^ concluded that the value of *Pedicularis* L was positively correlated with temperature. Our study found no significant correlations between the needle δ^13^C values of *P. armandii* and meteorological factors (Fig. 6). The δ^13^C values in middle-aged *P. yunnanensis* stands were significantly and positively correlated with temperature and mean surface temperature. This result indicates that the sensitivity of young and middle-aged *P. armandii* stands to temperature is lower than that of young *P. yunnanensis* stands because different species and age classes have different optimum photosynthetic temperatures.

Water also influences plant photosynthesis, respiration, and nutrient uptake and transport, as well as other biochemical processes, such as the synthesis of cell wall substances, proteins, and chlorophyll. It promotes the accumulation of sugars, proline, and other substances and affects the activity of some enzymes. If plants are water deficient, the photosynthetic capacity of the leaves decreases, and the δ^13^C value increases, thus increasing WUE^58^. Temperature and moisture are the dominant factors affecting plant photosynthesis and transpiration^59^. Moisture and temperature influence the atmospheric vapor pressure deficit, evapotranspiration, and mean relative humidity, affecting the leaf transpiration rate and stomatal conductance^60,61^. The correlation between the δ^13^C value and evapotranspiration, water vapor pressure, and mean relative humidity was highly significant for middle-aged *P. yunnanensis* forests. Only the mean air pressure was significantly negatively correlated with the δ^13^C value for young *P. yunnanensis* forests. This result indicates that temperature and moisture are the main factors affecting WUE in young and middle-aged stands of *P. yunnanensis* in China, and that temperature is the main factor driving the seasonal variation in δ^13^C of *P. yunnanensis* needles, with low δ^13^C values and low WUE at low temperatures.

## Conclusions

The water use strategies of young and middle-aged mixed stands of *P. armandii* and *P. yunnanensis* (typical timber species) in Yiliang area, Yunnan Province, were studied in different seasons by analyzing the needle δ^13^C values. The δ^13^C values of the two species were larger than those of typical subtropical species, and the two species showed high WUE. Young and middle-aged stands of *P. armandii* showed higher δ^13^C values (higher WUE), and young and middle-aged stands of *P. yunnanensis* showed lower δ^13^C values (lower WUE), indicating that *P. armandii* exhibited a more conservative water use strategy than *P. yunnanensis*. This difference was attributed to the genetic characteristics of the species.

The temperature, precipitation, and needle δ^13^C values were relatively high in summer and autumn in *P. yunnanensis* and *P. armandii* during the growing season. In contrast, in spring and winter, *P. armandii* needles had lower δ^13^C values only in spring and *P. yunnanensis* needles had lower δ^13^C values in spring and winter. Both species were affected by moisture and temperature. *P. yunnanensis* middle-aged forests were influenced by temperature and moisture during growth, and the temperature had a larger influence on the WUE. Moisture was the main factor affecting the WUE of young-aged *P. armandii* forests.

Global climate change has led to frequent occurrences of extreme climate events in recent years, such as droughts and high or low temperatures. As water and nutrient inputs decrease and high temperatures or prolonged cold periods increase, forestry production must find a balance between the growth rate, stress resistance, and productivity by adjusting the WUE. Thus, it is crucial to select site-specific tree species for afforestation. The results of this study can provide guidance for forestry production practices in central area of Yunnan province. However, this study has some limitations, we only analyzed the WUE of the two species for only one year. Thus, our results cannot be used for the water resource management of the two species because the soil water content has different spatial distributions in different seasons. In the future, we plan to investigate the water uptake from groundwater and the WUE of both species at different growth stages using stable isotope techniques (δ^18^O and δ^13^C) on a larger scale.

## Data Availability

The datasets used during the current study are available from the corresponding author on reasonable request.
